# Five new species of the *Stenus indubius* group (Coleoptera, Staphylinidae) from China

**DOI:** 10.3897/zookeys.165.1773

**Published:** 2012-01-13

**Authors:** Liang Tang, Li-Zhen Li

**Affiliations:** 1 Department of Biology, Shanghai Normal University, 100 Guilin Road, 1st Educational Building 323 Room, Shanghai, 200234 P. R. China

**Keywords:** Coleoptera, Staphylinidae, *Stenus indubius* group, identification key, new species, China

## Abstract

Five new species of the *Stenus indubius* group from China are described: *Stenus cangshanus* **sp. n.** from Yunnan Province, *Stenus hewenjiae* **sp. n.** from Sichuan Province, *Stenus taiyangshanus* **sp. n.** from Guangdong Province, *Stenus yinziweii* **sp. n.** and *Stenus zhaiyanbini* **sp. n.** from Guizhou Province.Diagnostic characters are illustrated and a key to the species of this group from the Chinese mainland is provided.

## Introduction

*Stenus indubius* group is a medium Asian group comprising 26 Japanese species (see Naomi 2006, also for a group definition) and ten Chinese species: *Stenus guniujiangense* Tang & Li, 2005 and *Stenus paradecens* Tang & Li, 2005 from Anhui Province, *Stenus zhuxiaoyui* Tang, 2008, *Stenus pectorifossatus* Tang, 2008 and *Stenus erlangshanus*Tang, 2008 from Sichuan Province, *Stenus hui* Tang & Puthz, 2009 from Shaanxi Province, *Stenus electristigma* Puthz, 2011, *Stenus shibatai* Puthz, 2011, *Stenus shibataianus* Puthz, 2011 and *Stenus shibataiellus* Puthz, 2011 from Taiwan.

All hitherto known members from the Chinese mainland of the group are brownish to blackish and brachypterous. In this paper, we describe five new species of the group collected in various mountainous areas in South China, three of them with orange spots on elytra, a character also present in Taiwanese species of this group Puthz (2011).

## Material and methods

The specimens examined in this paper were collected by sifting leaf litters in forests and killed with ethyl acetate. For examination of the male genitalia, the last three abdominal segments were detached from the body after softening in hot water. The aedeagi, together with other dissected pieces, were mounted in Euparal (Chroma Gesellschaft Schmidt, Koengen, Germany) on plastic slides. Photos of sexual characters were taken with a Canon G7 camera attached to an Olympus SZX 16 stereoscope; habitus photos were taken with a Canon macro photo lens MP-E 65 mm attached to a Canon EOS40D camera.

The type specimens treated in this study are deposited in the following public and private collections:

NMB Museum of Natural History Basel, Switzerland

NSMT National Museum of Nature and Science, Tokyo

SHNU Department of Biology, Shanghai Normal University, P. R. China

SMNS Staatliches Museum für Naturkunde Stuttgart, Germany

cKish private collection T. Kishimoto, Tokyo

cPut private collection V. Puthz, Schlitz, Germany

cSch private collection M. Schülke, Berlin

cSmet private collection A. Smetana, Ottawa

cWat private collection Y. Watanabe, Tokyo

The measurements of proportions are abbreviated as follows:

BL body length, measured from the anterior margin of the clypeus to the posterior margin of abdominal tergite X

FL forebody length, measured from the anterior margin of the clypeus to the apex of the elytra (apicolateral angle)

HW width of head including eyes

PW width of pronotum

EW width of elytra

PL length of pronotum

EL length of elytra, measured from humeral angle

SL length of elytral suture

## Taxonomy

### Key to the species of the Stenus indubius group from mainland China

**Table:** 

1	Pronotum without median longitudinal furrow; elytra with surface weakly uneven; punctation of pronotum and elytra well delimited; abdominal segments IV–VI with tergites and sternites completely fused without joint suture (*Hypostenus*)	2
–	Pronotum with median longitudinal furrow; elytra with surface very uneven; punctation of pronotum and elytra more or less rugose and confluent; abdominal segments IV–VI with tergites and sternites distinctly separated (*Hemistenus*)	3
2	Body size larger (BL: 4.3–4.8 mm), elytra shorter (EL/EW =0.86–0.93). Habitus: Fig. 2 in Tang and Li (2005); sexual characters: Figs 7–10 in Tang and Li (2005)	*Stenus guniujiangensis* Tang & Li, China: Anhui
–	Body size smaller (BL: 3.3–3.5 mm), elytra longer (EL/EW = 0.96–1.01). Habitus: Fig. 1 in Tang and Li (2005); sexual characters: Figs 3–6 in Tang and Li (2005)	*Stenus paradecens* Tang et Li, China: Anhui
3	Elytra bicolored with orange marks	4
–	Elyra unicolored without marks	6
4	Body size smaller (BL: 3.8 mm, FL: 1.8 mm); elytra with vague orange marks. Habitus: Figs 5, 6; sexual characters: Figs 33–39	*Stenus taiyangshanus* sp. n., China: Guangdong
–	Body size larger (BL ≥ 4.2 mm, FL ≥ 1.9 mm); elytral marks well delimited	5
5	Elytral marks larger, ranging from 3/5 to 4/5 as long as and 1/2 to 2/3 as broad as the respective elytron. Habitus: Figs 7, 8; sexual characters: Figs 40–50. BL : 4.3–4.7 mm	*Stenus yinziweii* sp. n., China: Guizhou
–	Elytral marks smaller, ranging from 1/3 to 1/2 as long as and 1/3 to 2/5 as broad as the respective elytron. Habitus: Figs 9, 10; sexual characters: Figs 51–61. BL : 4.2–5.1 mm	*Stenus zhaiyanbini* sp. n., China: Guizhou
6	Head broader, 1.24 times as wide as elytra; punctation of head especially in lateral portion sparser, where interstices may be a little larger than half the diameter of punctures; pronotum with short median longitudinal furrow and vorticose rugae. Habitus: Figs 3, 4; sexual characters: Figs 22–32. BL : 3.6–4.2 mm	*Stenus hewenjiae* sp. n., China: Sichuan
–	Head narrower, no more than 1.19 times as wide as elytra; punctation of head denser, interstices in lateral portion smaller than half the diameter of punctures; pronotum with short to very long median longitudinal furrow without distinct vorticose rugae	7
7	Pronotum with very long median longitudinal furrow extending along all of midline; elytra as long as wide; punctation of abdominal tergites III–VIII extremely dense. Habitus: Fig. 1 a in Tang et al. (2009); sexual characters: Fig 2 a, Figs 3 a–c in Tang et al. (2009). BL : 3.8–4.7 mm	*Stenus hui* Tang& Puthz, China: Shaanxi
–	Pronotum with median longitudinal furrow shorter, not extending along all of midline; elytra shorter than wide; punctation of abdominal tergites III–VII not extremely dense	8
8	Punctation of head denser; pronotal and elytral punctation less rugose and less confluent; elytral disc relatively even with less distinct impressions and suture slightly convex. Habitus: Figs 1, 2; sexual characters: Figs 11–21. BL : 3.6–4.2 mm	*Stenus cangshanus* sp. n., China: Yunnan
–	Punctation of head less dense; pronotal and elytral punctation more rugose and more confluent; elytral disc uneven with distinct, deep impressions and suture strongly convex	9
9	Abdominal punctation denser, interstices on abdominal tergites III–VII distinctly smaller than half the diameter of punctures; posterior margin of male abdominal sternite VII without emargination. Habitus: Fig 2 in Tang and Zhao (2008); sexual characters: Figs 9–13 in Tang and Zhao (2008). BL: 4.0–4.2 mm	*Stenus pectorifossatus* Tang, China: Sichuan
–	Abdominal punctation sparser, interstices on abdominal tergites III–VII smaller than half the diameter of punctures; posterior margin of male abdominal sternite VII with slight median emargination	10
10	Body larger (BL: 3.8–4.2 mm); median longitudinal furrow and impressions on pronotum very deep. Habitus: Fig 3 in Tang and Zhao (2008); sexual characters: Figs 14–18 in Tang and Zhao (2008)	*Stenus erlangshanus* Tang, China: Sichuan
–	Body smaller (BL: 3.0–3.7 mm); median longitudinal furrow and impressions on pronotum relatively shallow. Habitus: Fig 1 in Tang and Zhao (2008); sexual characters: Figs 4–8 in Tang and Zhao (2008)	*Stenus zhuxiaoyui* Tang, China: Sichuan

#### Stenus cangshanus

Tang & Li sp. n.

urn:lsid:zoobank.org:act:CA82E0DC-0E4E-4299-A495-4D8658871B53

http://species-id.net/wiki/Stenus_cangshanus

Figs 1, 2 11–21

##### Type material.

**Holotype. China: Yunnan:** male, glued on a card with labels as follows:“China: Yunnan Prov., Dali City, Cang Shan, alt. 2300 m, 10.VII.2010, Liang TANG Leg.” “Holotype / *Stenus cangshanus* / Tang & Li” [red handwritten label] (SHNU). **Paratypes.** 3 males and 5 females, same data as for the holotype (SHNU); 1 female, Dali, 1600–2000 m, 5–8.VII.1990, L. & M. Bocák (NMB); 1 female, Cangshan mountains, 25.38N, 100.09E, 2600–3100m, 5–6.VI.1993, Vít Kubán (NMB); 1 female, Dali, Cangshan mountains, 2700 m, 17.VII.1995, Bolm (NMB); 1 male, 1 female, Laohu Shan, 2200 m, Dali Shi, 3.IX.1992, Y. Watanabe (cWatanabe, cPut); 1 male, Zhonghe Feng 2200 m, Diancang Shan Mts., Dali Shi, 4.IX.1993, Y. Watanabe (cWatanabe); 1 male, Zhonghe Feng, 2540 m, Diancang Shan Mts, 28.X.1995, S. Uéno & N. Xiao (cWat); 1 female, above Dali, 2700–2900 m, 14.IV.1999, W. Schawaller (SMNS); 4 males, Dali Bai Nat. Aut: Pref., Diancang Shan, 4 km W Dali old town, 25°41.4'N, 100°06.7'E, 2900–3000 m, E slope with devasted forest and old pine forest, mushrooms, 31.VIII.2003, M. Schülke (C03–20) (cSch, cPut); 3 males, 3 females, ibidem, 31.VIII.2003, A. Smetana (C143) (cSmet, cPut); 1 male, 1 female, 3 km W Dali, Diancang Shan, 25°41.1'N, 100°06.8'E, 2600–2650 m, 30.VIII.2093, A. Smetana (C 141) (cSmet); 1 male, 3 km W Dali, Diancang Shan, 2750 m, 25°41.1N, 100°06.8'E, 1.IX.2003, A. Smetana (C 144) (cSmet).

##### Description.

Brachypterous; body blackish, anterior margin of labrum, antennae, maxillary palpi and legs yellowish brown.

BL: 4.2–4.8mm; FL: 2.0–2.2 mm.

HW: 0.84–0.94 mm, PL: 0.69–0.78 mm, PW: 0.66–0.73 mm, EL: 0.72–0.78 mm, EW: 0.77–0.87 mm, SL: 0.50–0.54 mm.

Head 1.07–1.12 times as wide as elytra; interocular area with deep longitudinal furrows, median portion convex, slightly extending beyond the level of inner eye margins; punctures round, partly confluent, slightly larger and sparser on median area than those near inner margins of eyes, diameter of large punctures about as wide as apical cross section of antennal segment II; interstices faintly reticulated, much smaller than half the diameter of punctures except those along the midline of the convex median portion, which may be as wide as diameter of punctures. Antennae, when reflexed, extending a little before posterior margin of pronotum; relative length of antennal segments from base to apex as 12.0: 7.5: 16.5: 10: 11: 7.5: 8: 5: 5.5: 6: 7.5. Paraglossa oval.

Pronotum 1.05–1.09 times as long as wide; disk uneven, with distinct median longitudinal furrow, two impressions in anterior half, transverse impression in the middle, and two impressions in posterior half; punctures rugose and confluent, of similar size as those of head; interstices reticulated, more or less smaller than half the diameter of punctures except those at the bottom of longitudinal furrow, which could be larger.

Elytra 0.89–0.93 times as long as wide, distinctly constricted at base; lateral margins, with slight concavity at about half, gently divergent posteriad; disk moderately uneven with distinct longitudinal humeral impression, distinct postero-lateral impression and long sutural impression, suture moderately convex; punctation and interstices similar to those of pronotum.

Legs with hind tarsi 0.72 times as long as hind tibiae, tarsomeres IV distinctly bilobed.

Abdomen cylindrical; distinct paratergites absent, but rudimentary lateral border present, tergites and sternites distinctly split at about posterior eighth; posterior margin of tergite VII with palisade fringe; punctures of abdominal tergites III–VIII round to elliptic, gradually becoming smaller posteriad; interstices smaller than half the diameter of punctures, with relatively faint microsculpture on tergites III–VII and distinct reticulation on tergites VIII–X.

Male. Sternite VII with inconspicuous emargination at middle of posterior margin and a shallow impression before it; sternite VIII (Fig. 11) with semi-circular emargination at middle of posterior margin;sternite IX (Fig. 12) with very long apicolateral projections, posterior margin less serrate; tergite X (Fig. 13) with posterior margin convex. Aedeagus (Figs 14, 15) robust; expulsion hooks (Fig. 17) relatively small; parameres extending a little beyond apex of median lobe, almost straight, swollen in apical third, with two groups of setae on inner side (Fig. 16): 5 apical setae and 9 subapical setae.

Female. Abdomen broader than that in male; sternite VIII (Fig. 18) inconspicuously prominent at middle of posterior margin; tergite X (Fig. 19) slightly broader than that of male; sclerotized spermatheca as in Figs 20, 21.

**Figures 1, 2.**
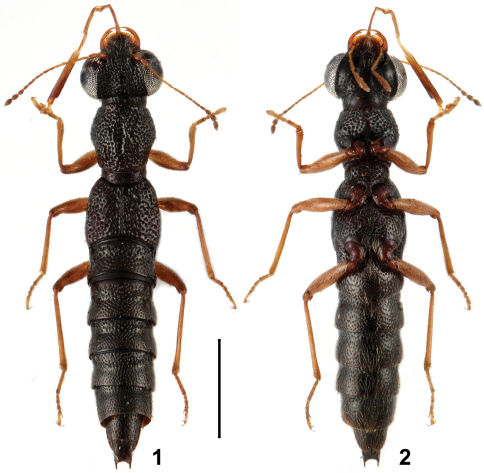
Habitus of *Stenus cangshanus* in dorsal and ventral view. Scale = 1 mm.

##### Distribution.

 China (Yunnan Province: Mt. Cang Shan).

##### Diagnoses.

 In general facies and body size, the new species resembles *Stenus hui* Tang, 2009, *Stenus pectorifossatus* Tang, 2008 and *Stenus erlangshanus* Tang, 2008, but it may be distinguished by the characters listed in the key, particularly by the different sexual characters.

##### Etymology

. The specific name is derived from “Cangshan”, the type locality of this species.

#### Stenus hewenjiae

Tang & Li sp. n.

urn:lsid:zoobank.org:act:0C303377-2124-4D17-BC89-7488C496EAFB

http://species-id.net/wiki/Stenus_hewenjiae

Figs 3, 4 22–32

##### Type material.

**Holotype. China: Sichuan:** male, glued on a card with labels as follows:“China: Sichuan Prov., Mt. Emei, Xixiangchi, alt. 2100 m, 29.VII.2009, He & Tang Leg.” “Holotype / *Stenus hewenjiae* / Tang & Li” [red handwritten label] (SHNU). **Paratypes.** 2 females, same data as for the holotype (SHNU); 1 female, Mt. Emei, Leidongping, 2400 m, 2.XI.1995, S. Uéno (cWat); 1 female, ibidem 2390 m, 4.X.1996, S. Nomura (NSMT); 1 male, ibidem 2310-2350 m, 5.X.1996, S. Nomura (NSMT); 1 female, Mt. Emei, above Xuedongping, 8.X.1997, T. Kishimoto (cKish).

##### Description.

Brachypterous; head blackish, labrum, pronotum, elytra and abdomen dark brown, anterior margin of labrum, antennae, maxillary palpi and legs yellowish brown.

BL: 3.6–4.2mm; FL: 1.8–2.1 mm.

HW: 0.83–0.90 mm, PL: 0.67–0.73 mm, PW: 0.58–0.70 mm, EL: 0.65–0.71 mm, EW: 0.67–0.77 mm, SL: 0.49–0.54 mm.

Head 1.18–1.24 times as wide as elytra; interocular area with deep longitudinal furrows, median portion convex, slightly extending beyond the level of inner eye margins; punctures round, mostly well delimited, slightly larger and sparser on median area than those near inner margins of eyes, diameter of large puncture about as wide as basal cross section of antennal segment II; interstices faintly reticulated, smaller than half the diameter of punctures except those along the midline of convex median portion and on the bottom of lateral furrows, which could be more or less larger. Antennae, when reflexed, extending a little before posterior margin of pronotum; relative length of antennal segments from base to apex as 10: 7.5: 15.5: 9.5: 9: 6: 6: 4: 4.5: 5: 7.5. Paraglossa oval.

Pronotum 1.05–1.13 times as long as wide; disk uneven, with distinct short median longitudinal furrow, transverse impression each in anterior half and in the middle, and two indistinct impressions in posterior half; punctures of similar size as those of head, rugose and confluent, forming vorticose rugae surrounding the longitudinal furrow; interstices indistinctly microsculptured, more or less smaller than half the diameter of punctures except those on the bottom of longitudinal furrow, which may be larger.

Elytra 0.92–0.97 times as long as wide, distinctly constricted at base, lateral margins with slight concavity in the middle, gently divergent posteriad; disk uneven with long deep longitudinal humeral impression and sutural impression, indistinct postero-lateral impression, suture strongly convex; punctation and interstices similar to those of pronotum.

Hind tarsi 0.69 times as long as hind tibiae, tarsomeres IV distinctly bilobed.

Abdomen cylindrical; distinct paratergites absent, but rudimentary lateral border present, tergites and sternites distinctly split at about posterior eighth; tergite VII with palisade fringe; punctures on abdominal tergites III–VIII round to elliptic, gradually becoming smaller posteriad; interstices on tergites III–VI faintly microsculptured and on tergites VIII–X distinctly microsculptured.

Male. Sternite VII with posteromedian portion slightly flattened; sternite VIII (Fig. 22) with semicircular emargination at middle of posterior margin; sternite IX (Fig. 23) with very long apicolateral projections, posterior margin serrate; tergite X (Fig. 24) with posterior margin truncate. Aedeagus (Figs 25, 26) slender; expulsion hooks (Fig. 28) large; parameres extending distinctly beyond apex of median lobe, bisinuate, folded at apical third, with about 25 setae on inner side (Fig. 27).

Female. Abdomen broader than that in male; sternite VIII (Fig. 29) inconspicuously prominent at middle of posterior margin; tergite X (Fig. 30) slightly emarginated at posterior margin; sclerotized spermatheca as in Figs 31, 32.

**Figures 3, 4.**
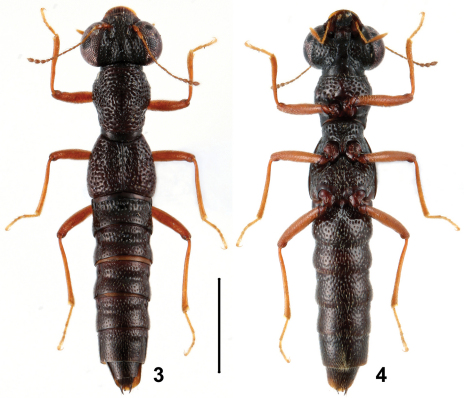
Habitus of *Stenus hewenjiae* in dorsal and ventral view. Scale = 1 mm.

##### Distribution.

China (Sichuan Province: Mt. Emei Shan).

##### Diagnoses.

This new species can be easily distinguished from allied species by the characters listed in key.

##### Comment.

 Dr. Puthz has a very similar (undescribed) species of the *indubius*-group from Mt. Emei, Leidongping.

##### Etymology.

 This species is named in honor of Ms. Wen-Jian He, wife of the first author, who collected some of the specimens of the new species.

#### Stenus taiyangshanus

Tang & Li sp. n.

urn:lsid:zoobank.org:act:1116E69D-64A5-45DC-990C-DC42E20BEEA1

http://species-id.net/wiki/Stenus_taiyangshanus

Figs 5, 6 33–39

##### Type material.

**Holotype. China: Guangdong:** male, glued on a card with labels as follows:“China: Guangdong Prov., Longmen County, Taiyangshan Mt., 16.VIII.2010, Liang Tang leg.” “Holotype / *Stenus taiyangshanus* / Tang & Li” [red handwritten label] (SHNU). **Paratype.** 1 female, same data as for the holotype (SHNU).

##### Description.

Brachypterous; body blackish, anterior margin of labrum, antennae, maxillary palpi and legs yellowish brown, each elytron with a vague elongate orange spot near lateral side.

BL: 3.8mm (the length of the immature female paratype with strongly contracted abdomen is not included); FL: 1.8 mm.

HW: 0.78–0.83 mm, PL: 0.62–0.67 mm, PW: 0.56–0.60 mm, EL: 0.64–0.67 mm, EW: 0.64–0.71 mm, SL: 0.45–0.48 mm.

Head 1.17–1.23 times as wide as elytra; interocular area with deep longitudinal furrows, median portion convex, slightly extending beyond the level of inner eye margins; punctures round, partly confluent, slightly larger and sparser on median area than those near inner margins of eyes, diameter of large punctures about as wide as basal cross section of antennal segment II; interstices faintly reticulated, much smaller than half the diameter of punctures except those on vertex and behind basiantennal tubercles, which may be much larger. Antennae, when reflexed, extending a little after posterior margin of pronotum; relative length of antennal segments from base to apex as 11: 7: 16: 8.5: 10: 7.5: 7: 5: 5.5: 5.5: 9. Paraglossa oval.

Pronotum 1.10–1.12 times as long as wide; disk slightly uneven, with distinct median longitudinal furrow, two indistinct impressions in anterior half, indistinct transverse impression in the middle, and two indistinct impressions in posterior half; punctures moderately rugose and confluent, of similar size as those of head; interstices, especially those on the bottom of median longitudinal furrow distinctly reticulated, more or less smaller than half the diameter of punctures except those on the bottom of median longitudinal furrow, which may be larger.

Elytra 0.95–1.01 times as long as wide, distinctly constricted at base, lateral margins with slight concavity at about half, gently divergent posteriad; disk slightly uneven with shallow longitudinal humeral impression, shallow postero-lateral impression and shallow sutural impression, suture moderately convex; punctation and interstices similar to those of pronotum.

Hind tarsi 0.7 times as long as hind tibiae, tarsomeres IV distinctly bilobed.

Abdomen cylindrical; distinct paratergites absent, but rudimentary lateral border present; tergite VII with palisade fringe; punctures on abdominal tergites III–VIII round to elliptic, gradually becoming smaller posteriad; interstices smaller to little larger than half the diameter of punctures, with relatively faint microsculpture throughout abdominal tergites.

Male. Sternite VII with posteromedian portion slightly flattened; sternite VIII (Fig. 33) with shallow emargination at middle of posterior margin; sternite IX(Fig. 34) with very long apicolateral projections, posterior margin serrate; tergite X (Fig. 35) with posterior margin slightly emarginated. Aedeagus (Figs 36, 37) robust, with setae at sclerotized apex of median lobe; expulsion hooks (Fig. 39) large; parameres extending distinctly beyond apex of median lobe, bisinuate, folded at apical fifth, with 21 setae on inner side (Fig. 38).

Female. Abdomen broader than that in male; sternite VIII inconspicuously prominent at the middle of posterior margin; tergite X slightly emarginated at posterior margin; sclerotized spermatheca can’t be observed in immature female and thus it can’t be illustrated here.

**Figures 5, 6.**
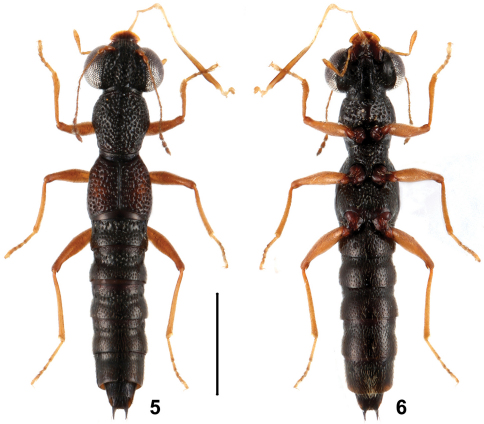
Habitus of *Stenus taiyangshanus* in dorsal and ventral view. Scale = 1 mm.

##### Distribution.

 China (Guangdong Province: Mt. Taiyang Shan).

##### Diagnoses.

This new species can be easily distinguished from related species by vague undelimited elytral spots and small body size.

##### Etymology.

 The specific name is derived from “Taiyangshan”, the type locality of this species.

#### Stenus yinziweii

Tang & Li sp. n.

urn:lsid:zoobank.org:act:74C97C73-989D-42FF-8019-22F10210EC09

http://species-id.net/wiki/Stenus_yinziweii

Figs 7, 8 40–50

##### Type material.

**Holotype. China: Guizhou:** male, glued on a card with labels as follows:“China: Guizhou Prov., Suiyang County, Kuankuoshui N. R., Gongtonggou, alt. 1530–1550m, 7–8.VI.2010, Lu, Yin & Zhai leg.” “Holotype / *Stenus yinziweii* / Tang & Li” [red handwritten label] (SHNU). **Paratypes.** 1 male and 6 females, same data as for the holotype (SHNU); 7 males and 12 females, same locality, 12–13.VI.2010, Lu, Yin & Zhai leg. (1 pair in cPut, rest in SHNU)

##### Description.

Brachypterous; body blackish, anterior margin of labrum, antennae, maxillary palpi and legs yellowish brown, each elytron with a large elongate orange spot, which is 3/5 to 4/5 as long as and 1/2 to 2/3 as broad as the respective elytron.

BL: 4.3–4.7mm; FL: 2.2–2.4 mm.

HW: 0.91–0.98 mm, PL: 0.73–0.82 mm, PW: 0.66–0.71 mm, EL: 0.75–0.83 mm, EW: 0.76–0.87 mm, SL: 0.52–0.55 mm

Head 1.10–1.20 times as wide as elytra; interocular area with deep longitudinal furrows, median portion convex, slightly extending beyond the level of inner eye margins; punctures round, partly confluent, slightly larger and sparser on median area than those near inner margins of eyes, diameter of large puncture about as wide as apical cross section of antennal segment II; interstices faintly reticulated, much smaller than half the diameter of punctures except those along the midline of convex median portion, which may be larger. Antennae, when reflexed, extending a little after posterior margin of pronotum; relative length of antennal segments from base to apex as 12: 7: 21: 10.5: 10.5: 8.5: 8.5: 6: 6: 6.5: 9. Paraglossa oval.

Pronotum 1.10–1.16 times as long as wide; disk moderately uneven, with distinct median longitudinal furrow, two shallow impressions in anterior half, shallow transverse impression in the middle, and two shallow impressions in posterior half; punctures slightly rugose and partially confluent, slightly larger than those on head; interstices, especially those on the bottom of median longitudinal furrow distinctly reticulated, more or less smaller than half the diameter of punctures except those on the bottom of median longitudinal furrow which may be much larger.

Elytra 0.95–0.98 times as long as wide, distinctly constricted at base, lateral margins with slight concavity at about half, gently divergent posteriad; disk uneven with distinct longitudinal humeral impression, distinct postero-lateral impression and long, deep sutural impression, suture convex; punctation and interstices similar to those of pronotum.

Hind tarsi 0.72 times as long as hind tibiae, tarsomeres IV distinctly bilobed.

Abdomen cylindrical; distinct paratergites absent, but rudimentary lateral border present, tergites and sternites split apically; tergite VII with palisade fringe; punctures on abdominal tergites III–VIII round to elliptic, gradually becoming smaller posteriad; interstices smaller than half the diameter of punctures, with relatively faint reticulation on tergites III–VII and distinct reticulation on tergites VIII–X.

Male. Sternite VII with posteromedian portion slightly flattened; sternite VIII (Fig. 40) with semicircular emargination at middle of posterior margin; sternite IX (Fig. 41) with very long apicolateral projections, posterior margin serrate; tergite X (Fig. 42) with posterior margin broadly rounded. Aedeagus (Figs 43, 44) slender; expulsion hooks (Fig. 46) relatively small; parameres extending a little beneath apex of median lobe, almost straight, with about 6 setae on inner side of apical portion (Fig. 45).

Female. Abdomen broader than that in male; sternite VIII (Fig. 47) inconspicuously prominent at middle of posterior margin; tergite X (Fig. 48) slightly emarginated at posterior margin; sclerotized spermatheca as in Figs 49, 50.

**Figures 7, 8.**
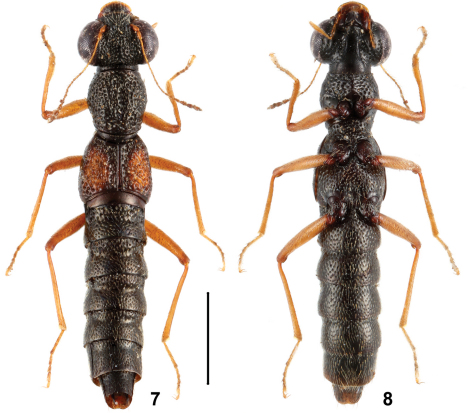
Habitus of *Stenus yinziweii* in dorsal and ventral view. Scale = 1 mm.

##### Distribution.

China (Guizhou Province : Kuankuoshui).

##### Diagnoses.

This new species closely resembles *Stenus zhaiyanbini* sp. n. and both of them live in the same nature reserve, but it may be distinguished from the latter species by larger size of the elytral marks and different sexual characters.

##### Etymology.

 This species is named in honor of Mr. Zi-Wei Yin, collector of the new species.

#### Stenus zhaiyanbini

Tang & Li sp. n.

urn:lsid:zoobank.org:act:5345D8F0-E45B-442D-8186-831F0F04C7AF

http://species-id.net/wiki/Stenus_zhaiyanbini

Figs 9, 10 51–61

##### Type material.

**Holotype. China: Guizhou:** male, glued on a card with labels as follows:“China: Guizhou Prov., Suiyang County, Kuankuoshui N. R., Baishagou, alt. 750–900m, 5.VI.2010, Yin & Zhai leg.” “Holotype / *Stenus zhaiyanbini* / Tang & Li” [red handwritten label] (SHNU). **Paratypes.** 9 males and 21 females, same locality, 2–5.VI.2010, Lu, Yin & Zhai leg. (1 pair in cPut, rest in SHNU)

##### Description.

Brachypterous; body blackish, anterior margin of labrum, antennae, maxillary palpi and legs yellowish brown, each elytron with a large oval orange spot, which is 1/3 to 1/2 as long as and 1/3 to 2/5 as broad as the respective elytron.

BL: 4.2–5.1 mm; FL: 1.9–2.4 mm.

HW: 0.83–1.03 mm, PL: 0.68–0.82 mm, PW: 0.60–0.74 mm, EL: 0.69–0.84 mm, EW: 0.69–0.88 mm, SL: 0.49–0.57 mm.

Head 1.14–1.21 times as wide as elytra; interocular area with deep longitudinal furrows, median portion convex, reaching the level of inner eye margins; punctures round, partly confluent, slightly larger and sparser on median area than those near inner margins of eyes, diameter of large punctures about as wide as apical cross section of antennal segment II; interstices hardly reticulated, much smaller than half the diameter of punctures except those along the midline of convex median portion, which may be larger. Antennae, when reflexed, extending a little after posterior margin of pronotum; relative length of segments from base to apex as 11.5: 7.5: 22: 12.5: 11: 9: 8.5: 6.5: 6.5: 6.5: 7.5. Paraglossa oval.

Pronotum 1.10–1.15 times as long as wide; disk uneven, with distinct median longitudinal furrow, two impressions in anterior half, transverse impression in about the middle, and two impressions in posterior half; punctures slightly rugose and partially confluent, slightly larger than those on head; interstices faintly reticulated, more or less smaller than half the diameter of punctures.

Elytra 0.94–1.01 times as long as wide, distinctly constricted at base, lateral margins, with slight concavity at about half, gently divergent posteriad; disk uneven with shallow longitudinal humeral impression, shallow postero-lateral impression and long, deep sutural impression, suture convex; punctation little larger than that of pronotum and interstices clearly microsculptured.

Hind tarsi 0.69 times as long as hind tibiae, tarsomeres IV distinctly bilobed.

Abdomen cylindrical; distinct paratergites absent, but rudimentary lateral border present, tergites and sternites split apically; tergite VII with palisade fringe; punctures on abdominal tergites III–VIII round to elliptic, gradually becoming smaller posteriad; interstices smaller than half the diameter of punctures, with relatively faint reticulation on tergites III–VII and distinct reticulation on tergites VIII–X.

Male. Sternite VII with posteromedian portion slightly flattened; sternite VIII (Fig. 51) with semicircular emargination in the middle of posterior margin; sternite IX (Fig. 52) with very long apicolateral projections, posterior margin serrate; tergite X (Fig. 53) with posterior margin slightly emarginated. Aedeagus (Figs 54, 55) slender; expulsion hooks (Fig. 57) relatively small; parameres extending a little beneath apex of median lobe, bended to inner side, with about 10 setae on inner side of apical portion (Fig. 56).

Female. Abdomen broader than that of male; sternite VIII (Fig. 58) inconspicuously prominent at middle of posterior margin; tergite X (Fig. 59) slightly emarginated at posterior margin; sclerotized spermatheca as in Figs 60, 61.

**Figures 9, 10.**
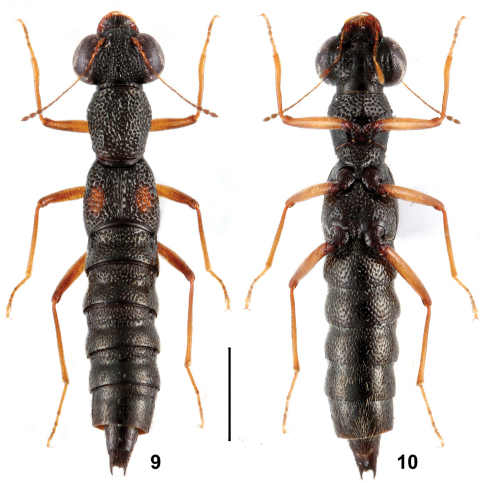
Habitus of *Stenus zhaiyanbini* in dorsal and ventral view. Scale = 1 mm.

##### Distribution.

China (Guizhou Province : Kuankuoshui).

##### Diagnoses.

This new species closely resembles *Stenus yinziweii* sp. n.in most aspects, except in smaller elytral marks and different sexual characters.

##### Etymology.

 The specific name is dedicated to Mr. Yan-Bin Zhai, collector of the new species.

#### 

**Figures 11–21.**
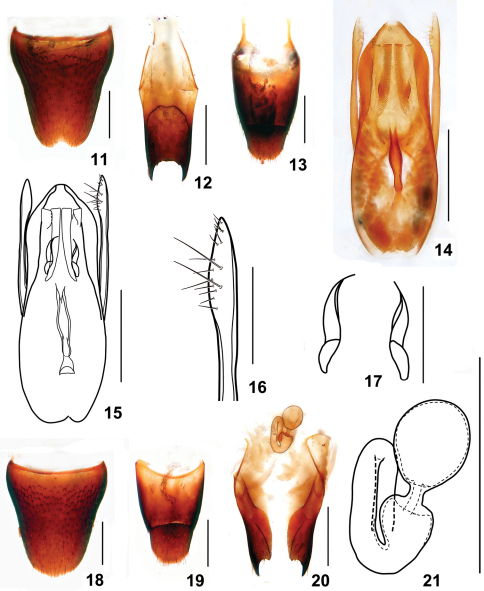
*Stenus cangshanus*. **11**male sternite VIII **12** male sternite IX **13** male tergites IX, X **14, 15** aedeagus **16** apical portion of paramere **17** expulsion hooks **18** female sternite VIII **19** female tergites IX, X **20** valvifers and spermatheca **21** spermatheca. Scales = 0.1 mm (**16, 17**), scales = 0.25 mm (**11–15, 18–21**).

**Figures 22–32.**
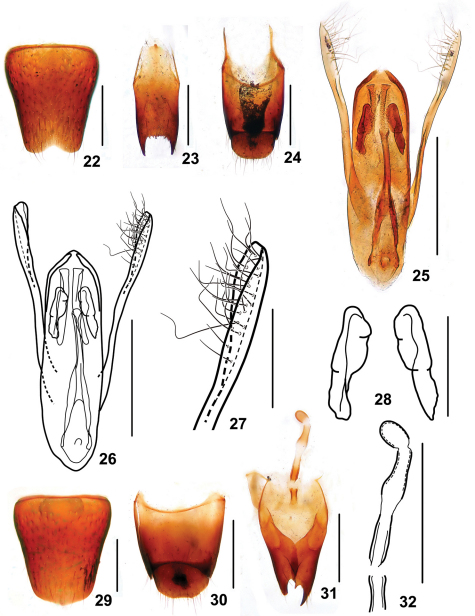
*Stenus hewenjiae*. **22** male sternite VIII **23** male sternite IX **24** male tergites IX, X **25, 26** aedeagus **27** apical portion of paramere **28** expulsion hooks **29** female sternite VIII **30** female tergites IX, X **31** valvifers and spermatheca **32** spermatheca. Scales = 0.1 mm (27, 28), scales = 0.25 mm (**22–26, 29–32**).

**Figures 33–39.**
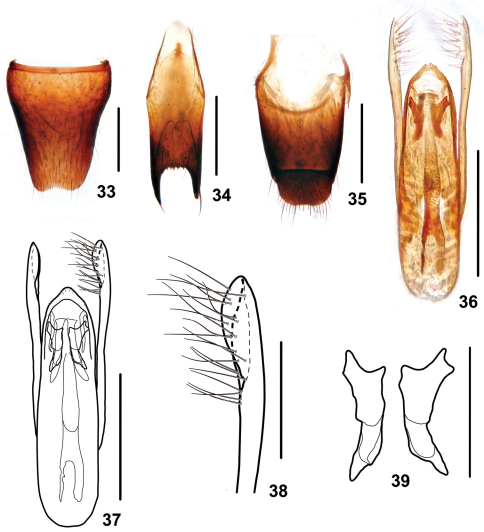
*Stenus taiyangshanus*. **33** male sternite VIII **34** male sternite IX **35** male tergites IX, X **36, 37** aedeagus **38** apical portion of paramere **39** expulsion hooks. Scales = 0.1 mm (38, 39), scales = 0.25 mm (**33–37**).

**Figures 40–50.**
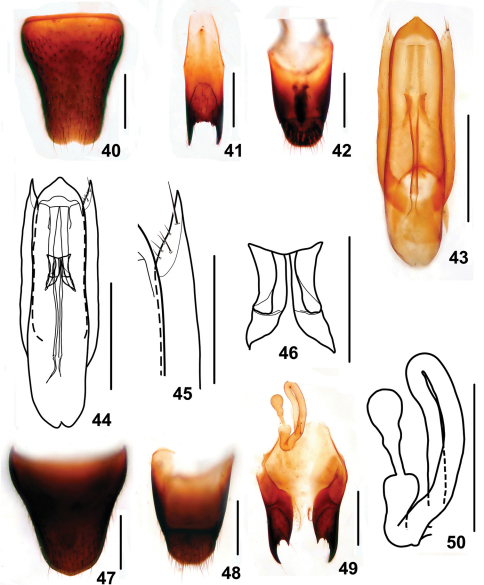
*Stenus yinziweii*. **40** male sternite VIII **41** male sternite IX **42** male tergites IX, X **43, 44** aedeagus **45** apical portion of paramere **46** expulsion hooks **47** female sternite VIII **48** female tergites IX, X **49** valvifers and spermatheca **50** spermatheca. Scales = 0.1 mm (**45, 46**), scales = 0.25 mm (**40–44, 47–50**).

**Figures 51–61.**
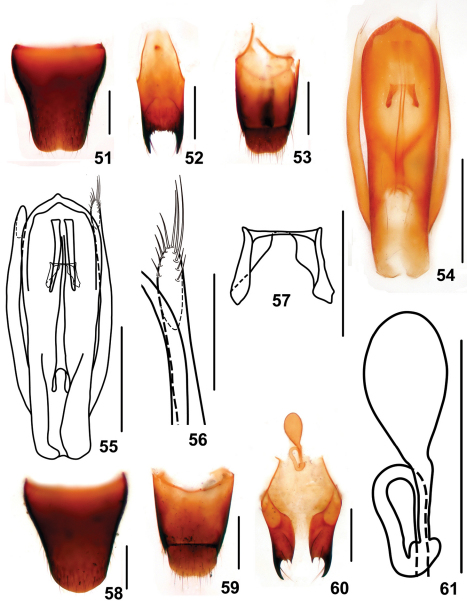
*Stenus zhaiyanbini*. **51** male sternite VIII **52** male sternite IX **53** male tergites IX, X **54, 55** aedeagus **56** apical portion of paramere **57** expulsion hooks **58** female sternite VIII **59** female tergites IX, X **60** valvifers and spermatheca **61** spermatheca. Scales = 0.1 mm (**56, 57**), scales = 0.25 mm (**51–55, 58–61**).

## Supplementary Material

XML Treatment for Stenus cangshanus

XML Treatment for Stenus hewenjiae

XML Treatment for Stenus taiyangshanus

XML Treatment for Stenus yinziweii

XML Treatment for Stenus zhaiyanbini
